# Combination of Naltrexone and Isotretinoin for the Treatment of Darier Disease

**DOI:** 10.7759/cureus.33321

**Published:** 2023-01-03

**Authors:** Tomás Costa, Clarisse Rebelo, Gabriela Marques Pinto, Bruno Duarte

**Affiliations:** 1 Dermatology, Centro Hospitalar Universitário Lisboa Central, Lisbon, PRT; 2 Dermatology, Centro Hospitalar Universitário do Algarve, Faro, PRT

**Keywords:** darier disease, dermatology case report, clinical dermatology, low-dose naltrexone, genodermatoses

## Abstract

Darier disease (DD) is an autosomal-dominant genodermatosis in which mutations in the ATP2A2 gene result in impaired intercellular adhesion and epidermal blistering. Treatment options usually rely on systemic retinoids, but a refractory disease is still a therapeutical challenge. Given the similarity of DD pathogenesis with Hailey-Hailey disease, concomitant treatment with low-dose-naltrexone (LDN) has been proposed. We present the case of a 34-year-old woman with a 20-year history of severe, biopsy-proven DD, previously treated with several unsuccessful topical and systemic treatments, including oral isotretinoin, cyclosporine, doxycycline, methotrexate, acitretin, and subcutaneous adalimumab. At presentation, she had widespread keratotic, crusted, brown papules on her trunk and proximal extremities. Treatment with oral LDN (4.5 mg/day in manipulated tablets) was then initiated while maintaining the current isotretinoin therapy. After three months, there was a nearly complete clearance of the lesions, and no adverse effects were reported.

## Introduction

Darier disease (DD) is an autosomal-dominant genodermatosis in which mutations in the ATP2A2 gene result in the dysfunction of a calcium ATPase pump (SERCA2 pump) [1]. The result is a chronic depletion of calcium levels in the endoplasmic reticulum, leading to impaired intercellular adhesion and epidermal blistering. Patients typically present with keratotic, crusted, red-brownish papules that favor seborrheic areas [2]. Systemic retinoids can be used, but treatment options are limited in refractory disease.

## Case presentation

We present the case of a 34-year-old woman with severe Darier disease who was referred to our dermatology department. The first skin lesions started when she was 12 years old and were initially located on the anterior surface of the trunk. However, over the years, they had spread to the face and upper and lower limbs.

She had been previously treated with several unsuccessful topical and systemic treatments, including oral cyclosporine, doxycycline, methotrexate, acitretin, and subcutaneous adalimumab. She was currently under oral isotretinoin at 0.5 mg/kg/day but still presented with disseminated lesions. She had no other known conditions, and there was no familiar history of similar cases. The patient reported worsening of the lesions during periods of high emotional stress, but no additional triggers were identified.

**Figure 1:**
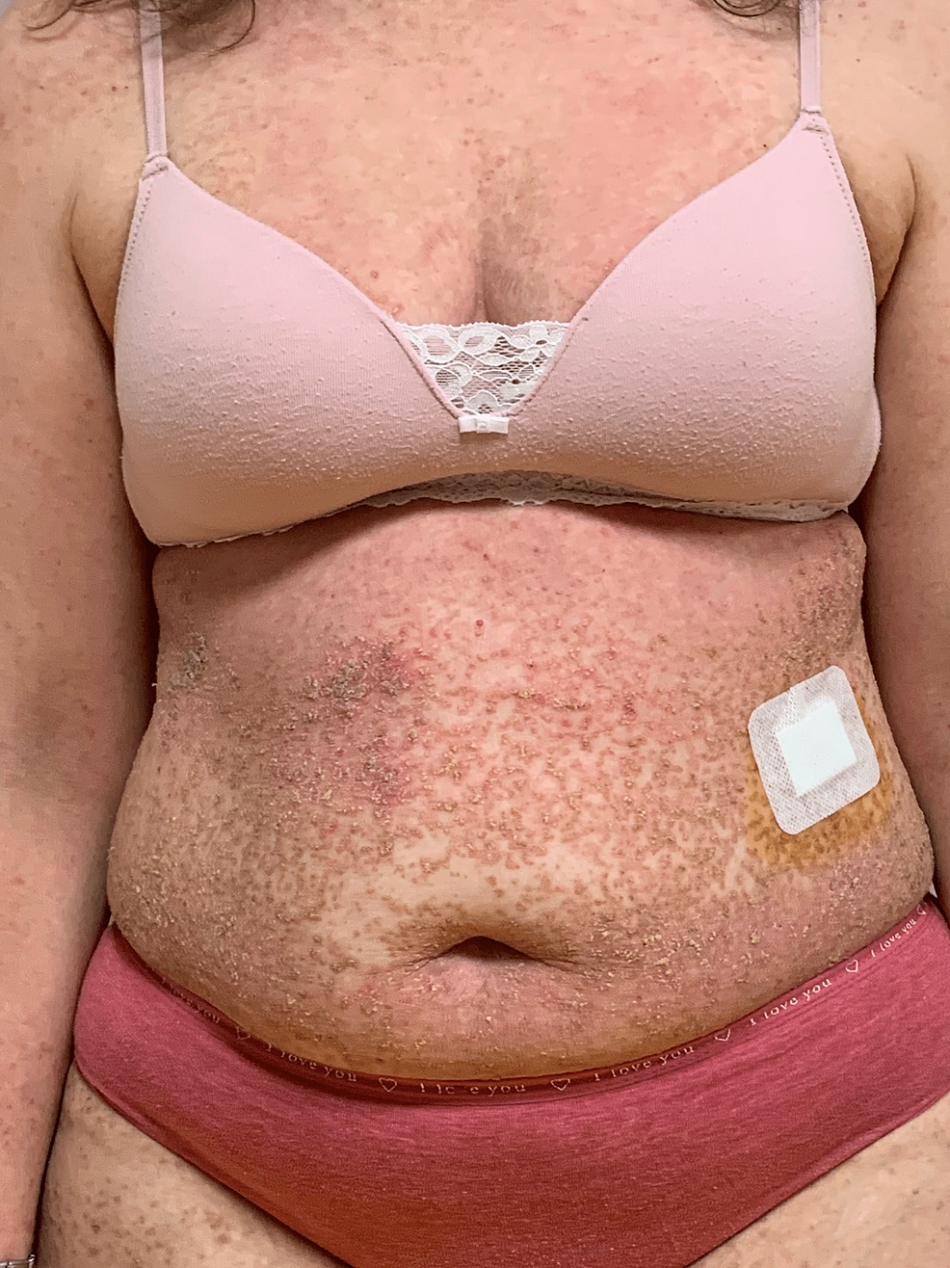
Patient presenting with severe Darier disease (baseline)

At presentation, she had widespread keratotic, crusted, brown papules on her trunk and proximal extremities (Figure 1), compatible with Darier disease.

**Figure 2:**
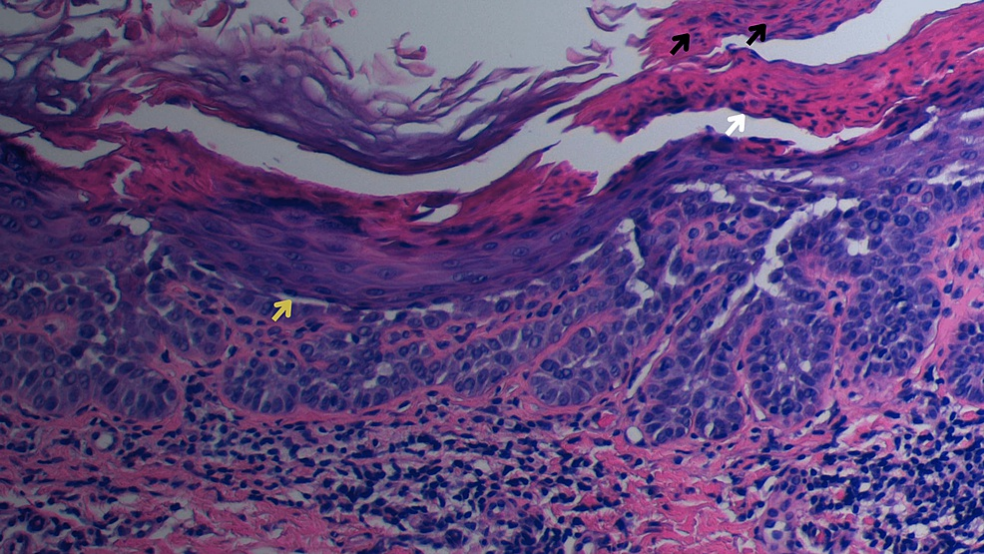
Histopathology of an abdominal papule, revealing acantholytic dyskeratosis A suprabasal cleft can be observed (yellow arrow). A rounded cell, with basophilic nuclei, perinuclear halo, and eosinophilic cytoplasm (white arrow), corresponds to the traditionally described "corps ronds." The elongated, small, acantholytic cells located in the upper layers of the stratum corneum (black arrows) correspond to the "grains."

A punch biopsy of an abdominal papule was then performed (Figure 2), revealing acantholytic dyskeratosis with a suprabasal cleft. Some rounded cells with basophilic nuclei, perinuclear halo, and eosinophilic cytoplasm were also evident, mainly located in the lower layers of the epidermis; and some elongated, small, acantholytic cells were located in the upper layers of the stratum corneum. These two types of cells represented the classically described “corps ronds and grains” pattern of Darier disease, thus confirming the diagnosis.

The case was discussed in our dermatology department weekly meeting, and given the lack of response to isolated systemic treatment with retinoids, the patient was started on low-dose naltrexone (4.5 mg/day, using manipulated oral tablets) while maintaining the current isotretinoin therapy.

**Figure 3:**
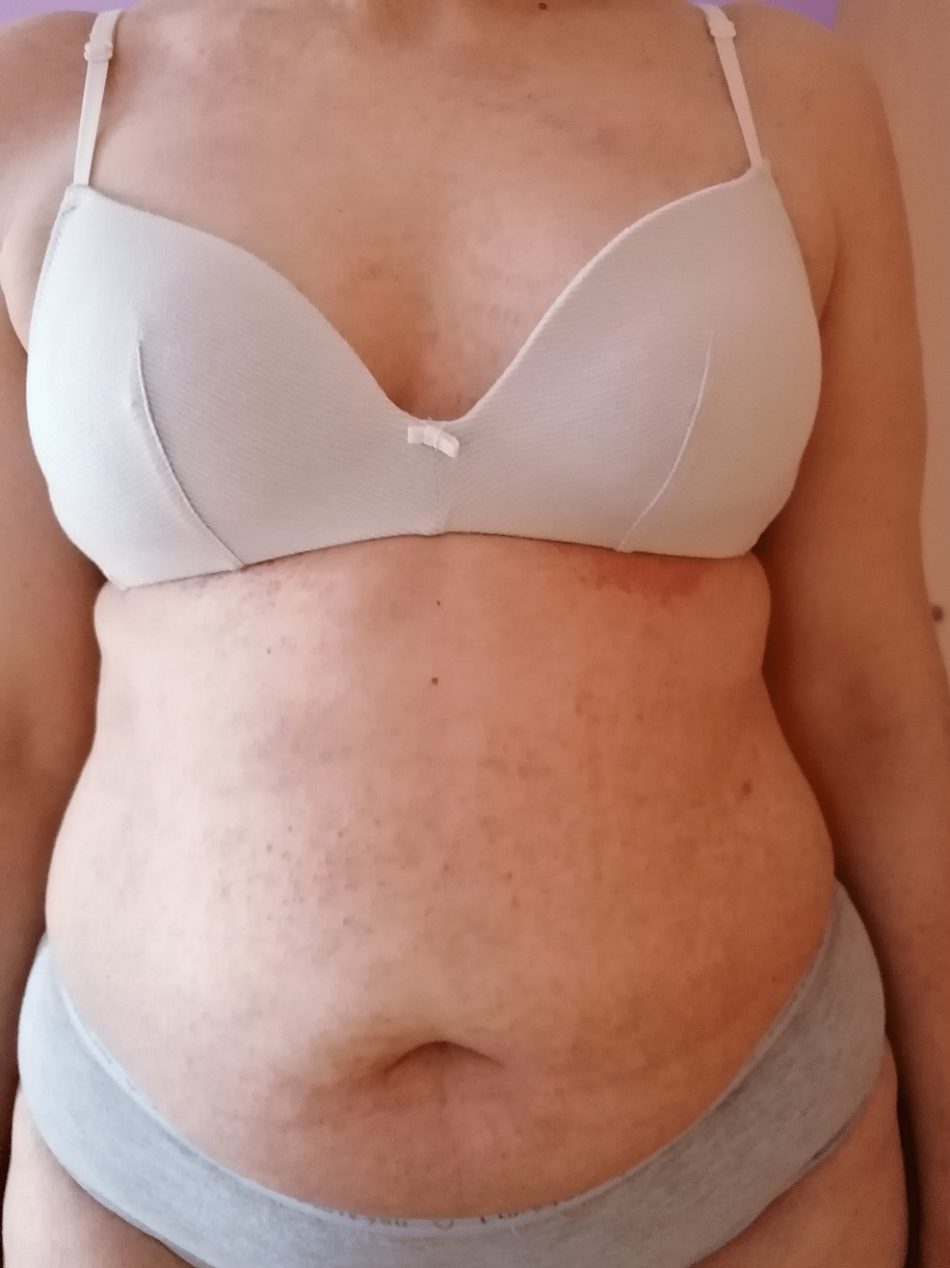
Patient after three months of combination therapy with LDN and oral isotretinoin LDN: low-dose-naltrexone

At the three-month follow-up, there was a nearly complete clearance of the lesions (Figure 3), and no adverse effects were reported.

## Discussion

LDN has been successfully used in some patients with Hailey-Hailey disease (HHD) [3]. This genodermatosis is similarly caused by defective calcium transport and homeostasis. Naltrexone is an µ-opioid receptor antagonist with minimal side effects and the potential for dependence.

It is unclear why LDN is beneficial in both DD and HHD, but it is known that the dysregulation of intracellular calcium signaling via defective ATPase pumps is the basis of both HHD and DD pathogenesis and that LDN can change intracellular calcium trafficking. In fact, by acting as a µ-opioid receptor antagonist, naltrexone negates its inhibitory action on transient receptor potential melastatin 3 (TRPM3)-dependent calcium influx, thus restoring the normal function of these calcium channels [4]. On the other hand, LDN also exerts immunoregulatory effects through various pathways, most notably as an antagonist of the nonopioid toll-like receptor 4 [5]. As such, several proinflammatory cytokines are downregulated by this inhibition, which might mitigate the overlapping inflammatory aspects of HHD and DD.

The use of LDN in DD was first described by Boehmer et al. [6] but with disappointing results. In their study, six patients with biopsy-proven DD were treated with LDN 4.5 mg and magnesium 200 mg once daily. Four patients had severe DD and showed no sustained clinical improvement. However, they have also described a very promising response in a patient that was still taking acitretin (25 p.o.) and postulated that systemic retinoids might have a synergistic effect with LDN.

In our case, adding naltrexone while maintaining oral isotretinoin led to a dramatic improvement in DD with a lack of noticeable side effects. As such, the therapy will be maintained indefinitely until it fails or becomes associated with some adverse effect that contraindicates its maintenance. These results seem to reinforce what was proposed by Boehmer et al. [6].

As such, and given the lack of effective treatments, LDN seems to present as a safe, inexpensive, and promising therapy for DD, which should be further explored in additional prospective trials.

## Conclusions

Darier disease is an autosomal-dominant genodermatosis with limited treatment options in cases refractory to systemic retinoids. We report a clinical case where concomitant treatment with low-dose naltrexone was associated with a dramatic improvement in a previously refractory patient. We expect this case report can lead to further prospective studies that can validate our approach.
